# Comparison of in‐field secondary cancer risk based on therapeutic proton dose between proton arc therapy and intensity‐modulated proton therapy in lung cancer patients

**DOI:** 10.1002/acm2.70793

**Published:** 2026-09-10

**Authors:** Yuankang Xu, Wenguang Chen, Junxiang Wu, Hao Guo, Fanrui Zeng, Tianyu Zhang, Feng Yang, Xinhui Huang, Da Zhang, Xianliang Wang

**Affiliations:** ^1^ School of Physics University of Electronic Science and Technology Chengdu China; ^2^ Department of Radiation Oncology Precision Radiation in Oncology Key Laboratory of Sichuan Province Sichuan Clinical Research Center for Cancer Sichuan Cancer Center, Sichuan Cancer Hospital & Institute, Affiliated Cancer Hospital of University of Electronic Science and Technology of China Chengdu China; ^3^ The College of Nuclear Technology and Automation Engineering Chengdu University of Technology Chengdu China; ^4^ Institute of Automatic and Molecular Physics Sichuan University Chengdu China

**Keywords:** intensity‐modulated proton therapy, lung cancer, proton arc therapy, secondary cancer risk

## Abstract

**Background:**

Proton arc therapy (PAT) is an emerging delivery modality that delivers proton beams through continuous gantry rotation combined with spot scanning. Compared with intensity‐modulated proton therapy (IMPT), PAT improves target conformity and spares organs at risk. However, the dynamic multi‐angle irradiation pattern may alter the low‐dose region distribution: the low‐dose bath expands while the integral dose decreases. The net effect on secondary cancer risk, especially in radiosensitive lungs, remains unclear. Quantitative risk assessment is essential for clinical translation of PAT.

**Purpose:**

To quantitatively compare secondary lung cancer risk between PAT and IMPT using organ equivalent dose (OED) and lifetime attributable risk (LAR) models, and to assess cross‐population robustness across China, France, Sweden, and Germany.

**Methods:**

Data from 25 lung cancer patients (60 Gy/30 fractions) were retrospectively included. For each patient, robustly optimized IMPT and PAT plans were generated with a 3 mm setup and 3% range uncertainties across 21 error scenarios. Dosimetric parameters—ipsilateral lung $V_{5}$, $V_{20}$, and mean dose; whole lungs $V_{5}$, $V_{20}$, and mean dose; and esophageal mean dose—were compared between the two techniques. The mechanistic OED model (with repopulation factor R) and its simplified linear model were used to calculate OED for the ipsilateral lung, both lungs, and the esophagus. LAR was computed by integrating excess absolute risk over the remaining lifetime using patient age, sex, and population‐specific parameters (China, France, Sweden, Germany). Relative risk was assessed via the LAR ratio (IMPT/PAT). Paired t‐tests or Wilcoxon tests were used with *p* < 0.05.

**Results:**

Under the linear model, PAT produced statistically markedly but modest reductions in absolute LAR for lungs (*p* < 0.001). Under the mechanistic model, reductions were markedly only in certain populations: ipsilateral lung in France (*p* = 0.001), Sweden (*p* < 0.001), Germany (*p* = 0.003); whole lungs in France (*p* = 0.046) and Sweden (*p* = 0.014); but not in China (*p* = 0.083; *p* = 1.000) or Germany for whole lungs (*p* = 0.317). No marked esophageal differences (*p* > 0.05). LAR ratios were consistent across countries (ipsilateral lung: 1.07–1.09; esophagus: 1.04–1.13), demonstrating robustness.

**Conclusions:**

PAT does not increase the modeled in‐field secondary cancer risk in the lungs compared with IMPT and shows comparable risk for the esophagus. The risk reduction trend is robust across Chinese and European populations. These findings suggest that PAT does not increase modeled secondary lung cancer risk compared with IMPT and may even provide a modest risk reduction, providing quantitative evidence for clinical application.

## 1 INTRODUCTION

Proton therapy, by virtue of its unique Bragg peak physical characteristics, can substantially reduce the irradiated volume and integral dose to normal tissues surrounding the tumor as compared with photon therapy.^1^ Proton therapy continues to evolve, with proton arc therapy (PAT) emerging as a novel delivery modality. PAT achieves dynamic multi‐angle irradiation through continuous gantry rotation combined with spot‐scanning, offering more degrees of freedom for beam incidence compared with conventional spot‐scanning intensity‐modulated proton therapy (IMPT) and thus demonstrating potential advantages in dose distribution. Studies have shown that PAT can achieve superior dose conformity while maintaining delivery efficiency.^1^, ^2^ Furthermore, PAT has the potential to optimize linear energy transfer (LET) distributions, which may influence its relative biological effectiveness, suggesting potential advantages in dosimetry, biological effectiveness, and delivery efficiency.

However, this dynamic, multi‐angle irradiation pattern may also alter the distribution characteristics of low‐dose regions: the low‐dose bath is spread over larger volumes, albeit with a reduced integral dose and improved target conformality.^3^ Although the long‐term risk of secondary cancer associated with low‐dose radiation is considered low, the impact of changes in its spatial extent and pattern on this risk remains unclear.^4^ This is particularly concerning for lung cancer patients, as the lung is a highly radiosensitive organ; low‐dose irradiation of the lung contributes to radiation‐induced pulmonary injuries such as pneumonitis and fibrosis,^5^, ^6^ and may also elevate the long‐term risk of secondary lung malignancies.^7^, ^8^ Nevertheless, the net effect of PAT on secondary cancer risk depends on the balance between the expanded low‐dose bath and the reduced intermediate to high‐dose exposure to organs at risk—the latter being a more established contributor to carcinogenesis. With the continuous improvement in cancer patient survival, the importance of managing treatment‐related late toxicities is increasingly recognized. Among these late effects, radiotherapy‐induced secondary cancer has drawn considerable attention due to its appreciable cumulative incidence, long latency period, and severe clinical consequences. Bartkowiak et al.^9^ reported an annual secondary cancer incidence of approximately 1%, predominantly occurring within or at the margins of the original radiation fields. Given the large population of long‐term cancer survivors, the absolute number of patients affected is substantial, and secondary cancers are often more difficult to treat with a poorer prognosis.^10^ Therefore, maximizing the reduction of long‐term radiation‐induced cancer risk while maintaining effective primary tumor control has become an important objective in the optimization and evaluation of modern radiotherapy regimens.^11^

Earlier theoretical modeling studies have predicted the potential of PAT in reducing secondary cancer risk.^2^ Subsequently, the organ equivalent dose (OED) model developed by Schneider et al.^12^, which incorporates the mechanism of cellular repopulation, has successfully quantified the carcinogenic risk of non‐uniform dose distributions, providing a reliable tool for comparing different radiotherapy techniques.^13^ Modeling studies based on this framework have demonstrated that IMPT can markedy reduce secondary cancer risk compared with photon radiotherapy.^12^ However, direct comparisons of secondary cancer risk between PAT and IMPT remain scarce. We hypothesized that, with superior high‐dose conformity, PAT may further reduce intermediate‐ and high‐dose exposure to organs at risk, thereby potentially yielding additional risk reduction benefits.

This study compared the ipsilateral lung and the whole lung $V_{5}$, $V_{20}$, and the mean dose, as well as the esophageal mean dose, between PAT and IMPT plans. Subsequently, based on the OED and lifetime attributable risk (LAR) models, we quantitatively compared the secondary cancer risk between PAT and IMPT plans in a cohort of lung cancer patients, aiming to provide data and theoretical support for the clinical translation and risk‐benefit assessment of PAT in lung cancer, with the explicit caveat that this comparison is restricted to therapeutic‐dose‐related in‐field risks and does not include potential contributions from secondary neutrons. Considering that baseline cancer incidence, age structure, and life expectancy differ across countries, the LAR ratio (LAR_IMPT_/LAR_PAT_) may vary geographically. Therefore, we performed simulated calculations using life tables and risk model parameters from four countries—China, France, Sweden, and Germany—to assess the cross‐population stability of the risk reduction effect of PAT.

## 2 METHODS

### 2.1 Patient information

This study was a retrospective analysis that included 25 patients with pathologically confirmed lung cancer who received conventional photon radiotherapy between January 2024 and July 2025. Among them, 22 were male, and 3 were female. The original treatment employed a conventional fractionation regimen with a prescription dose of 60 Gy delivered in 30 fractions. The basic patient characteristics are shown in Table 1. The requirement for informed consent was waived due to the retrospective nature of this study. The study was approved by the institutional ethics committee.

**TABLE 1:** Characteristics of the patients.

Characteristics		No
Sex	Male	22
	Female	3
T stage	T1	3
	T2	6
	T3	5
	T4	11
	Median	Range
Age (years)	65	59–70
CT interval time (days)	29	20–34
CTV (pCT, cm^3^)	31.99	17.94–56.56
CTV (aCT, cm^3^)	18.01	11.55–43.28

### 2.2 Treatment planning

Both IMPT and PAT plans were designed and optimized based on the original photon radiotherapy plans to ensure that the dosimetric and secondary cancer risk comparisons between the two techniques were conducted under identical anatomical structures and dose objectives. All proton treatment plans were generated using the Proteus^+^PLUS proton therapy system (IBA, Louvain‐la‐Neuve, Belgium) and were optimized in the RayStation treatment planning system (version 7.0, RaySearch Laboratories, Stockholm, Sweden). The same optimization conditions and dose constraints for organs at risk (OARs) were applied to both IMPT and PAT plans. The OAR dose constraints were adopted from the National Comprehensive Cancer Network (NCCN) Clinical Practice Guidelines for Non‐Small Cell Lung Cancer and were as follows:  spinal cord $D_{max}\leq$4500 cGy**;** esophagus $D_{\max}\leq 105\%$ of the prescription dose, $\;D_{mean}\leq$3400 cGy, $V_{50}\;\leq 50\%;$ heart $V_{40}\leq 20\%$, $D_{mean}\leq$ 2000 cGy and lungs $V_{20}\leq 35\%-40\%,$ $D_{mean}\leq 2000$ cGy. IMPT plans employed multi‐field optimization, with 3 to 4 beam angles individually customized for each patient based on tumor location and the distribution of surrounding OARs.^14^ PAT plans used a discrete beam optimization simulation method with a beam spacing of 10°, incorporating 13 to 17 fields per plan and similarly applying multi‐field optimization.^15^

Dose calculations were performed using the Monte Carlo algorithm, with the beam uncertainty parameter set to 0.5%. All plans underwent robust optimization, accounting for a setup uncertainty of 3 mm and a range uncertainty of 3%. A total of 21 scenarios were evaluated, including 1 nominal scenario, 6 setup shifts, 2 range shifts, and 12 combined setup and range error scenarios.

### 2.3 Secondary cancer risk calculation

The OED model, established and extensively validated by Schneider et al.^16^, was employed to quantify secondary cancer risk.^17^

The linear model is based on the simplest assumption that the excess risk of cancer induction in an irradiated tissue is directly proportional to the locally absorbed physical dose. Under this assumption, the risk equivalent dose (RED) is numerically equal to the physical dose D:

(1) $$RED\;\left(D\right)=D$$

The OED for the entire organ is then computed as the volume‐weighted average of RED across all voxels:

(2) $$OED=\frac{1}{V_{T}}\sum_{i}V\left(D_{i}\right)RED\left(D_{i}\right)$$

where $V_{T}$ is the total organ volume, and $V(D_{i})$ is the organ sub‐volume receiving dose Di. Under the linear model, the OED is numerically equal to the mean absorbed dose of the organ.

The mechanistic model employs a linear‐quadratic framework to describe the RED^12^, ^13^:

(3) $$\begin{array}{cc} & RED\;\left(D\right)=\frac{e^{-D\alpha^{\prime }}}{R\alpha^{\prime }}\;\left[1-2R+R^{2}e^{D\alpha^{\prime }}-{\left(1-R\right)}^{2}e^{\frac{DR\alpha^{\prime }}{1-R}}\right],\\ & \;\mathrm{where}\;\alpha^{\prime }=\beta \frac{D}{D_{T}}d_{T} \end{array}$$

The repopulation/repair factor R reflects the tissue's ability to repair damage through cellular repopulation after irradiation; a value closer to 1 indicates a stronger repair capacity and a less pronounced risk saturation effect in high‐dose regions. The linear coefficient α and quadratic coefficient β are key biological parameters describing radiation‐induced cellular damage. In this study, the R and α values for the lung and esophagus were derived from fitting cohort data of Hodgkin lymphoma radiotherapy patients.^18^ Although these parameters originate from a Hodgkin lymphoma cohort, they represent organ‐specific radiation sensitivity and repopulation capacity rather than disease‐specific effects, and have been widely applied in secondary cancer risk modeling for various primary tumor sites.^18^ β was derived assuming an $\frac{\alpha }{\beta }$ ratio of 3 Gy for each organ. The total prescription dose $D_{T}$ and fraction dose $d_{T}$ in this study were 60 and 2 Gy, respectively.

After calculating the $\mathrm{RED}(D_{i})$ for each voxel, the OED under the mechanistic model was obtained through the same volume‐weighted summation using Equation (2).

Subsequently, the excess absolute risk (EAR) and LAR were calculated to quantify the absolute risk faced by individual patients.

EAR represents the additional cancer incidence attributable to radiotherapy at a specific attained age (agea), where agex denotes the age at exposure (i.e., the patient's age at treatment).

(4) $$\begin{array}{cc} & EAR\left(D,agea,agex,s\right)=\beta_{EAR}\;\;OED\left(D\right)\\ & exp\left[\gamma_{e}\left(agex-30\right)+\gamma_{\alpha }\ln\left(\frac{agea}{75}\right)\right]\times \left(1\pm 0.17\right),\\ & +\;\mathrm{for}\;\mathrm{females},\;-\mathrm{for}\;\mathrm{males} \end{array}$$

Here, the initial slope of the linear no‐threshold model $\beta_{\mathrm{EAR}}$, and the age‐modifying parameters $\gamma_{e}$ and $\gamma_{\alpha }$, vary depending on the population background. Chinese population parameters were based on data from ICRP Publication 103^19^. Parameters for the French, Swedish, and German populations were derived using the same source transferred to European populations (ERR–EAR weighting method). Due to the absence of sex‐specific baseline cancer incidence and age‐modifying parameters for these European populations in the source data, sex‐averaged parameters were applied. Specific values are provided in Table 2. The α and R values for esophageal parameters were taken from Paganetti et al.^20^ and remained consistent across countries. The initial slope $\beta_{\mathrm{EAR}}$ represents the excess cancer incidence per unit OED, differing by organ and sex. The age‐modifying parameters $\gamma_{e}$ and $\gamma_{\alpha }$ quantify the effects of age at exposure (i.e., age at radiotherapy) and attained age on risk, respectively. To account for sex‐based risk differences, a sex adjustment factor (multiply by 1.17 for males,0.83 for females) was applied as recommended by the International Commission on Radiological Protection.^19^

**TABLE 2:** Parameters used for risk calculation.

Country	Organ	Sex	$\beta_{\mathrm{EAR}}$	$\gamma_{e}$	$\gamma_{\alpha }$	α	β	*R*	Note
China	Lung	Male	6.47	0.001	4.25	0.042	0.0140	0.83	
		Female	8.97						
	Esophagus	Male	0.48	0.064	2.38	0.026	0.0087	0.81	*α* and R from Paganetti et al. (2020)^20^
		Female	0.66						
France/ Sweden/ Germany	Lung		8.00	0.002	4.23	0.042	0.0140	0.83	
	Esophagus		3.20	−0.002	1.9	0.026	0.0087	0.81	*α* and R from Paganetti et al. (2020)^20^

The core parameters of the risk models used in this study are listed in the table 2 below:

Finally, the LAR represents the cumulative risk of developing a secondary cancer due to the radiotherapy course,^16^ obtained by integrating the EAR from the age at exposure (agex) to the life expectancy (agea) and weighting by the conditional probability of survival from agex to agea, $\frac{S(agea)}{S(agex)}$:

(5) $$\begin{array}{cc} & LAR\;(D,agea,agex,s)\\ & =\int_{agex}^{agea}EAR\left(D,agea,agex,s\right)\;\frac{S\left(agea\right)}{S\left(agex\right)}da \end{array}$$

The life expectancies used for LAR calculation, obtained from life tables published by Chinese and European Union agencies,^21^, ^22^ are listed below in table 3:

**TABLE 3:** Life expectancy by sex for each country.

Country	Male	Female
China	75.37	80.88
France	80.2	85.8
Sweden	82.3	85.3
Germany	78.9	83.5

### 2.4 Statistical analysis

The data collected in this study comprised three main categories: patient clinical characteristics, treatment planning dosimetric variables, and derived variables based on the risk models. Clinical characteristics, such as age, sex, T stage, and clinical target volume, were descriptive variables used to characterize the baseline features of the study population. Dosimetric variables, extracted from IMPT and PAT plans, included $V_{5}$, $V_{20}$ and mean dose for the ipsilateral lung, whole lungs, and esophagus, and were used to directly compare dose distribution differences between the two techniques. Derived variables were the analytical variables computed through the risk models, including OED and LAR for each organ.

All data analyses were performed using SPSS software (v26.0, IBM Corp, Armonk, NY, USA). Paired‐samples t‐tests or two‐tailed non‐parametric Wilcoxon signed‐rank tests were employed. When the differences of the paired data followed a normal distribution, the paired t‐test was used; otherwise, the paired two‐tailed non‐parametric Wilcoxon signed‐rank test was applied. A p‐value < 0.05 was considered statistically markedly. All results are presented as mean ± standard deviation (mean ± SD).

## 3 RESULTS

### 3.1 Plan dosimetric evaluation

**FIGURE 1:**
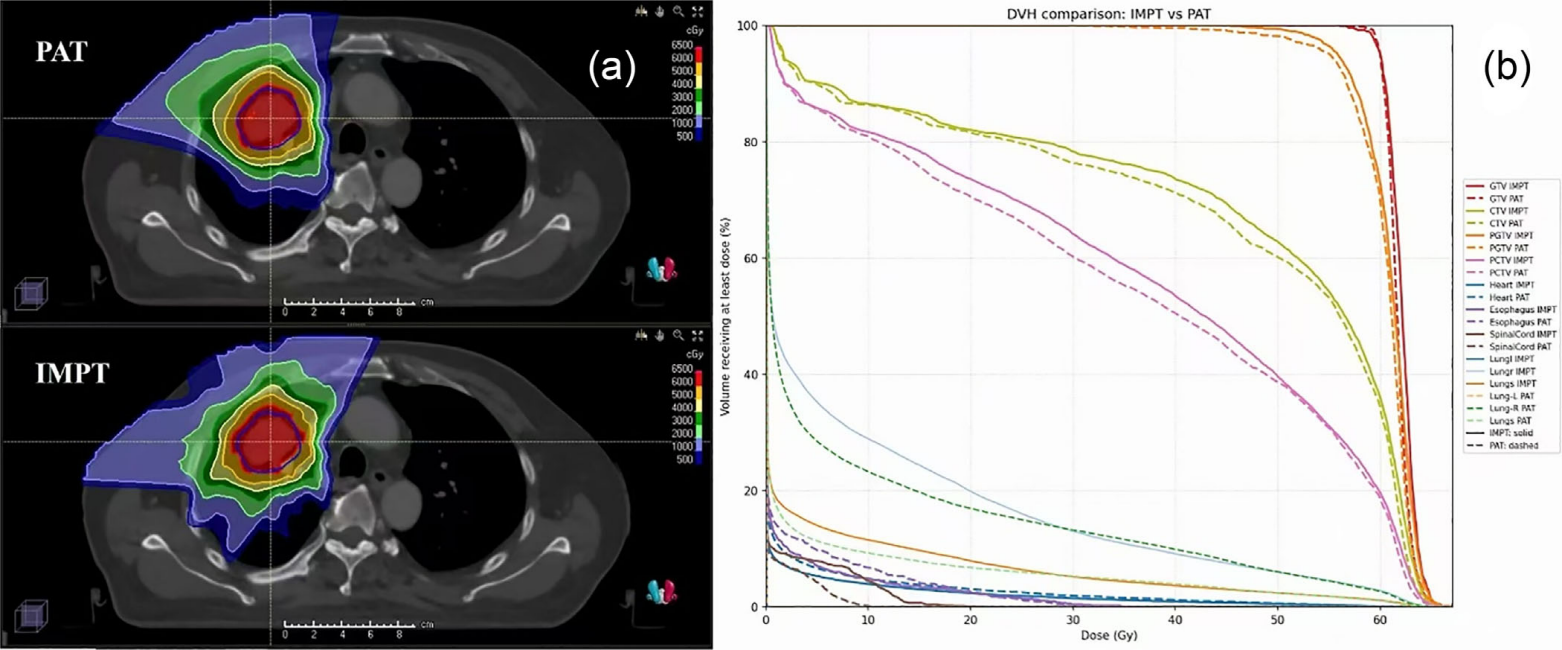
Comparison of dose distributions and dose‐volume histograms (DVH) between IMPT and PAT for a representative lung cancer patient. (a) Coronal views of the dose distributions. The IMPT plan (bottom) shows limited‐angle irradiation with low‐dose regions (blue) extending along beam paths, whereas the PAT plan (top) achieves steeper dose gradients and better conformity to the clinical target volume (CTV, red) through continuous gantry rotation. (b) Corresponding DVH curves for the CTV, planning target volume (PTV), and organs at risk (heart, esophagus, spinal cord, lungs). Solid lines represent IMPT, and dashed lines represent PAT. PAT demonstrates reduced intermediate‐ and low‐dose volumes for the ipsilateral and contralateral lungs, consistent with the more dispersed low‐dose pattern observed in (a). Both plans met the clinical dose constraints for all organs at risk.

Although all IMPT and PAT plans met clinical requirements for target coverage and OAR sparing, the coronal dose distributions of a representative case (Figure 1) illustrate the differences in dose deposition patterns. The IMPT plan showed limited‐angle irradiation, with low‐dose regions (blue) closely related to beam paths. In contrast, PAT, with continuous gantry rotation, provided a steeper dose gradient and better high‐dose conformity to the CTV (red). Notably, the PAT plan confines low‐dose irradiation more to the ipsilateral lung, indicating that dynamic multi‐angle irradiation can better “disperse” low‐dose radiation over larger non‐critical volumes, thereby reducing sustained low‐dose radiation exposure to these sensitive organs.

The dosimetric evaluation of all proton plans in this study referred to the normal tissue dose–volume constraints for conventionally fractionated chemoradiotherapy from the National Comprehensive Cancer Network (NCCN) Clinical Practice Guidelines for Non‐Small Cell Lung Cancer.^14^ As shown in Table 4, the organ‐at‐risk doses for both PAT and IMPT plans were substantially below the guideline‐recommended constraint limits. PAT plans demonstrated a consistent trend of superiority over IMPT plans in key metrics, such as the ipsilateral lung $V_{5}$, $V_{20}$ and mean dose; this systematic reduction in low‐dose regions may be associated with a reduced secondary cancer risk, although this remains to be confirmed clinically. Regarding low‐dose exposure to organs at risk, PAT plans generally showed a more favorable trend. As presented in Table 4, for the ipsilateral lung, the mean dose in PAT plans (797.0 cGy) was lower than that in IMPT plans (846.0 cGy). Corresponding low‐dose volumetric parameters, such as the ipsilateral lung $V_{5}$ and $V_{20}$, were also reduced by 2.5% and 1.4%, respectively, in PAT plans. The dose to the whole lungs exhibited a similar trend.

**TABLE 4:** DVH metrics for IMPT and PAT.

Organ	Metric	Units	Constraint	IMPT(x̄ ± SD)	PAT(x̄ ± SD)	*P*
Ipsilateral lung	$V_{5}$	%	≤60%	27.43±7.46	24.98±7.79	<0.001
Ipsilateral lung	$V_{20}$	%	≤35%	16.42±6.05	15.00±5.70	<0.001
Ipsilateral lung	Mean	cGy	≤1500	846.00±292.81	796.99±293.96	<0.001
Lungs	$V_{5}$	%	≤70%	12.46±2.85	11.38±3.14	<0.001
Lungs	$V_{20}$	%	≤40%	7.45±2.17	6.81±2.10	<0.001
Lungs	Mean	cGy	≤2000	384.57±103.81	362.49±106.57	<0.001
Esophagus	Mean	cGy	≤3400	178.61±270.92	176.56±261.50	<0.001

### 3.2 Comparison of organ equivalent dose

To quantify the dose‐attributable risk induced by the two treatment techniques in each organ at risk, we calculated the OED values for the ipsilateral lung, whole lungs, and esophagus under both the linear and mechanistic models, using the OED ratio ($\mathrm{OE}D_{\mathrm{IMPT}/\mathrm{PAT}}$) as the relative risk metric. A ratio greater than 1 suggests a lower risk with PAT compared with IMPT, and vice versa.

**FIGURE 2:**
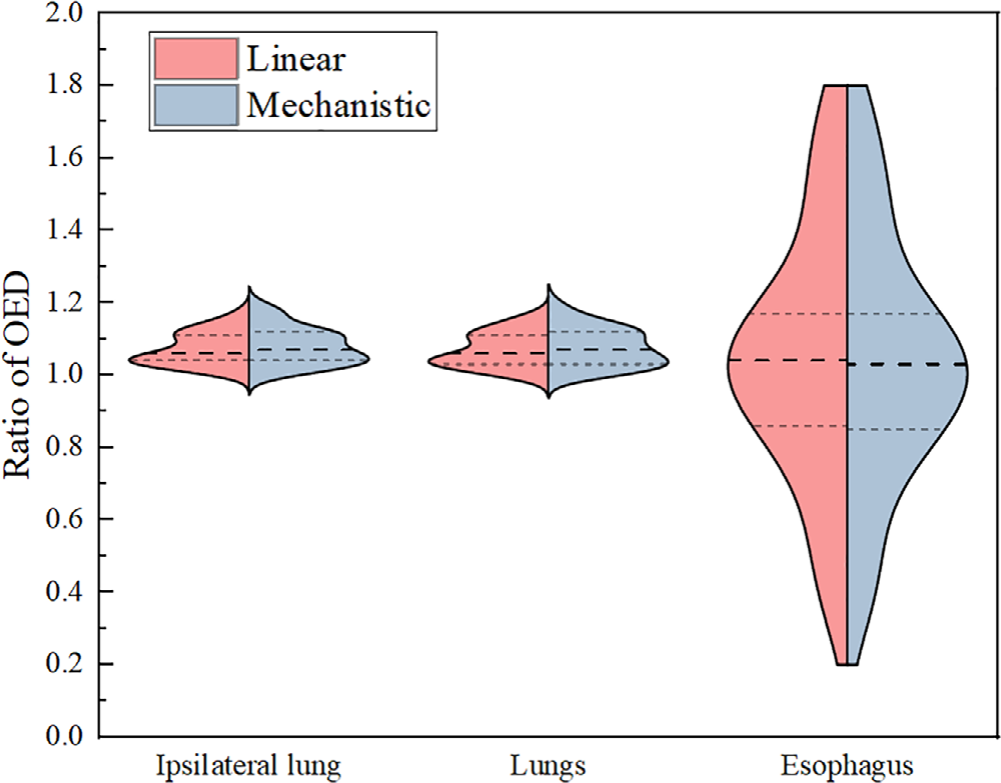
$\mathrm{OE}D_{\mathrm{IMPT}/\mathrm{PAT}}$ for the ipsilateral lung, whole lungs, and esophagus under the linear and mechanistic models. Values > 1 favor PAT.

The split‐violin plot in Figure 2 displays the distribution characteristics of the OED ratios for each organ. The OED ratio distributions for the ipsilateral lung and whole lungs were highly concentrated, with mean values consistently around 1.07 to 1.08, indicating a uniform risk reduction trend for PAT in these two organs. In contrast, the OED ratio distribution for the esophagus was more dispersed; although some patients exhibited ratios below 1, the mean values (linear model 1.05 ± 0.36, mechanistic model 1.04 ± 0.34) remained slightly greater than 1, suggesting that any potential risk reduction with PAT for the esophagus was not uniform across all patients. Paired statistical tests comparing OED values between PAT and IMPT for each organ showed that the differences did not reach statistical significance under either the linear or mechanistic model (all *p* > 0.05).

**FIGURE 3:**
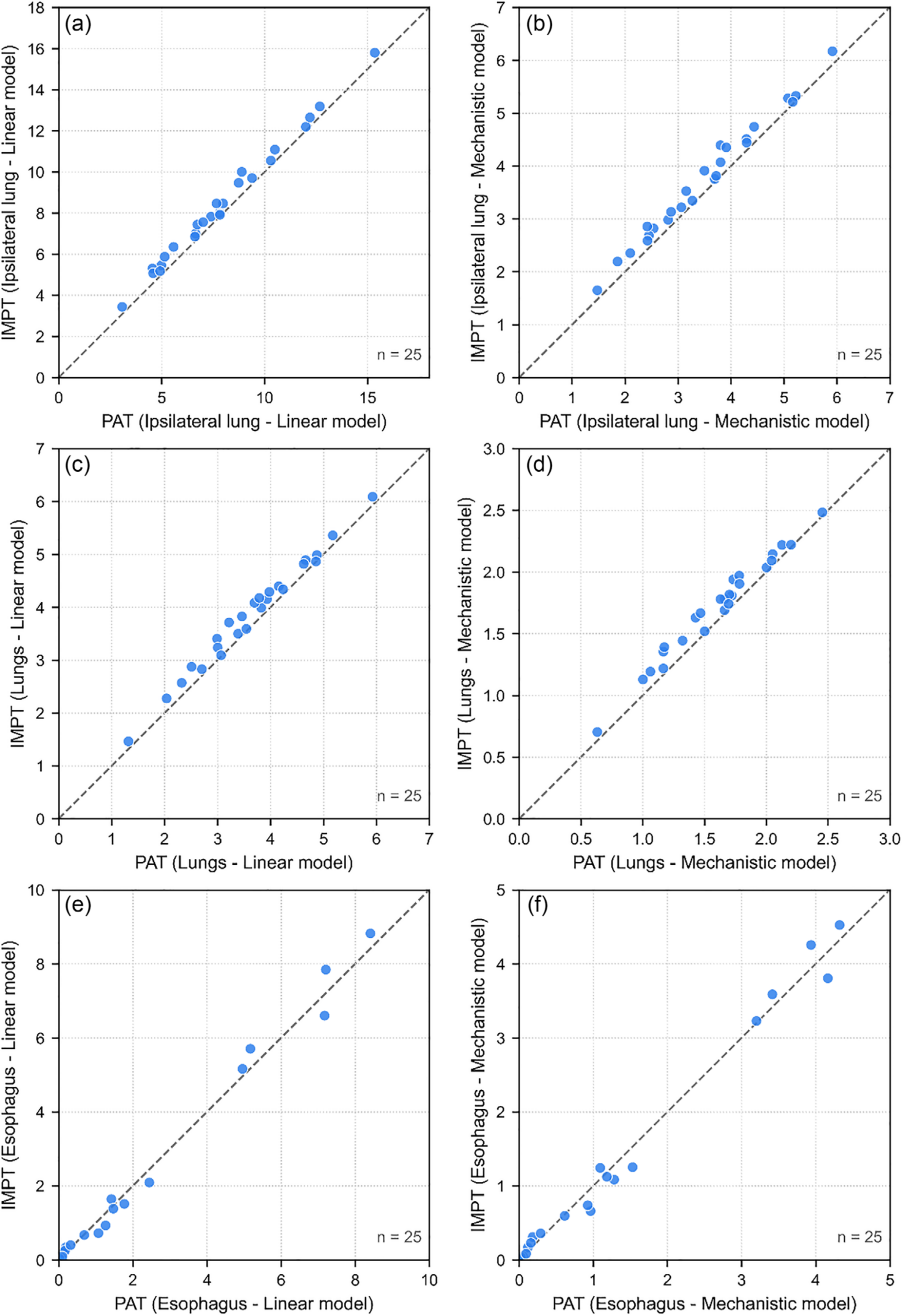
Patient‐level scatter plots of $\mathrm{OE}D_{\mathrm{IMPT}}$ versus $\mathrm{OE}D_{\mathrm{PAT}}$ across three organs and two risk models. Each panel displays the individual patient distribution: (a) ipsilateral lung—linear model; (b) ipsilateral lung—mechanistic model; (c) whole lungs—linear model; (d) whole lungs—mechanistic model; (e) esophagus—linear model; (f) esophagus—mechanistic model. The x‐axis represents OED under PAT, and the y‐axis represents OED under IMPT. The dashed diagonal line is the unity line (y = x); points above the line indicate a lower OED (i.e., lower modeled risk) with PAT compared with IMPT, while points below the line indicate a lower OED with IMPT. Clustering of most points near or slightly above the unity line reflects the modest overall differences between the two techniques. Notably, the esophagus panels (E and F) show wider scatter across both sides of the unity line compared with the lung panels, confirming the substantial inter‐patient heterogeneity and the absence of a consistent risk advantage for PAT at the individual level for this organ.

As shown in Figure 3, the esophagus exhibited the widest distribution of OED ratios (ranging from approximately 0.2 to 1.8), indicating substantial inter‐patient heterogeneity. Although the maximum reduction for a single patient was ∼1.8, the median ratio was close to 1, and some patients showed ratios below 1, implying a potential increased risk with PAT in those individuals. Thus, no consistent or pronounced risk advantage for the esophagus can be inferred. Specific data are presented in Table 5.

**TABLE 5:** Comparison of OED values (mean ± SD) for three organs under the linear and mechanistic models, along with the OED ratio (IMPT/PAT). The Ratio column represents $\mathrm{OE}D_{\mathrm{IMPT}}$ divided by $\mathrm{OE}D_{\mathrm{PAT}}$; a value greater than 1 indicates a lower modeled risk with PAT. The *p* values refer to comparisons between PAT and IMPT within each model. All OED values are expressed in cGy.

Organ	Units		OED‐linear(x̄± SD)				OED‐mechanistic(x̄± SD)		
		IMPT	PAT	Ratio	*p*	IMPT	PAT	Ratio	*p*
Ipsilateral lung	cGy	8.44 ± 2.95	7.94 ± 2.97	1.07 ± 0.04	<0.001	3.74 ± 1.12	3.49 ± 1.14	1.08 ± 0.05	<0.001
Lungs	cGy	3.87 ± 1.04	3.65 ± 1.06	1.07 ± 0.05	<0.001	1.72 ± 0.41	1.61 ± 0.42	1.08 ± 0.05	<0.001
Esophagus	cGy	1.79 ± 2.71	1.77 ± 2.61	1.05 ± 0.36	<0.001	1.10 ± 1.49	1.11 ± 1.47	1.04 ± 0.34	<0.001

The corresponding OED ratios (IMPT/PAT) under the linear model were 1.063 ± 0.075 for the ipsilateral lung, 1.061 ± 0.051 for the whole lungs, and 1.047 ± 0.356 for the esophagus. Under the mechanistic model, the ratios were 1.071 ±0.074,1.064±0.050,and1.043± 0.335, respectively (data not shown in the table).

Table 5 lists the comparison of absolute OED values for the three organs under the two models. For the ipsilateral lung, the mean OED of PAT was reduced by approximately 5.9% under the linear model and by 6.7% under the mechanistic model compared with IMPT. The whole lungs exhibited a similar trend, with PAT reducing the mean OED by approximately 5.7% and 6.4% under the linear and mechanistic models, respectively. The esophageal OED showed minimal differences between the two techniques: the mean IMPT and PAT values were 1.79 and 1.77 cGy under the linear model, and 1.10 and 1.11 cGy under the mechanistic model, nearly identical.

### 3.3 Risk comparison based on lifetime attributable risk

Building on the OED analysis, we further incorporated population‐specific epidemiological parameters (baseline cancer incidence, life expectancy, etc.) for China, France, Sweden, and Germany to calculate the LAR and its ratio ($\mathrm{LA}R_{\mathrm{IMPT}/\mathrm{PAT}}$), to evaluate the absolute benefit of the PAT risk reduction effect in real populations and its cross‐population robustness.

Specific data are provided in Table 6:

**TABLE 6:** Comparison of LAR values (in %) for three organs in four countries under the linear and mechanistic models. All values are expressed as percentages.

			LAR‐Linear(x̄ ± SD)			LAR‐Mechanistic(x̄ ± SD)		
Organ	Units	Country	IMPT	PAT	*p*	IMPT	PAT	*p*
Ipsilateral lung	%	China	5.85 ± 3.78	5.51 ± 3.66	<0.001	2.59 ± 1.53	2.41 ± 1.45	0.083
		France	10.80 ± 5.05	10.06 ± 4.92	<0.001	4.79 ± 2.01	4.41 ± 1.90	0.001
		Sweden	12.48 ± 5.36	11.75 ± 5.29	<0.001	5.53 ± 2.11	5.16 ± 2.04	<0.001
		Germany	8.93 ± 4.51	8.40 ± 4.41	<0.001	3.95 ± 1.80	3.69 ± 1.72	0.003
Lungs	%	China	2.60 ± 1.25	2.45 ± 1.22	0.008	1.16 ± 0.54	1.08 ± 0.51	1.000
		France	4.79 ± 1.55	4.51 ± 1.56	0.008	2.13 ± 0.64	1.99 ± 0.64	0.046
		Sweden	5.61 ± 1.67	5.29 ± 1.70	0.025	2.50 ± 0.69	2.33 ± 0.69	0.014
		Germany	3.99 ± 1.42	3.76 ± 1.42	0.025	1.77 ± 0.60	1.66 ± 0.58	0.317
Esophagus	%	China	0.53 ± 0.75	0.54 ± 0.77	0.317	0.33 ± 0.43	0.35 ± 0.45	0.317
		France	0.76 ± 1.15	0.72 ± 1.09	0.317	0.48 ± 0.68	0.46 ± 0.67	0.317
		Sweden	0.86 ± 1.30	0.83 ± 1.24	0.157	0.54 ± 0.77	0.53 ± 0.75	1.000
		Germany	0.66 ± 1.01	0.64 ± 0.96	1.000	0.42 ± 0.61	0.41 ± 0.59	0.317

**FIGURE 4:**
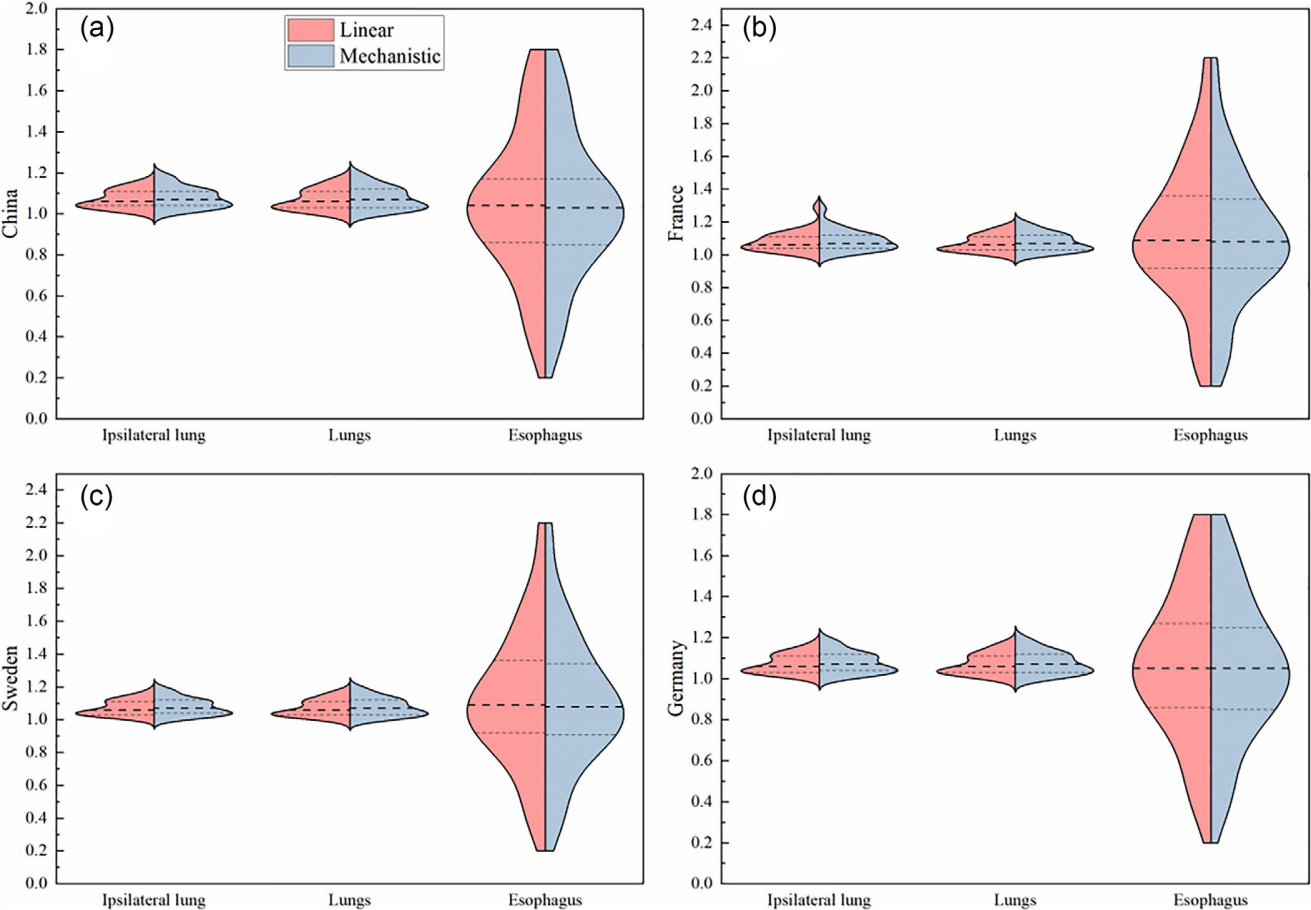
Boxplots of the LAR ratio (IMPT/PAT) for the ipsilateral lung across four populations under the linear and mechanistic models. (a) China, (b) France, (c) Sweden, and (d) Germany. The boxes represent the interquartile range (Q1–Q3), the central horizontal line indicates the median, and the whiskers extend to the minimum and maximum values (excluding outliers). A ratio greater than 1 indicates a lower modeled secondary cancer risk with PAT compared with IMPT. Across all countries, the median ratios are consistently close to or slightly above 1.0 under both models, with the mechanistic model showing a slightly wider interquartile range. The inset values in panels C and D denote extreme data points beyond the whiskers. These results demonstrate that the risk reduction trend for PAT is generally consistent across populations, although the magnitude is modest and inter‐patient variability exists.

Table 6 summarizes the absolute LAR values and inter‐group comparisons for each organ across the four populations. Taking the Chinese population as an example, the LAR of the ipsilateral lung under the linear model was 5.85% for IMPT and 5.51% for PAT, and under the mechanistic model was 2.59% and 2.41%, respectively. The whole lungs and esophagus showed similar trends. Notably, although the absolute LAR values differed considerably across countries due to variations in baseline cancer incidence and life expectancy (e.g., the ipsilateral lung LAR in the French population was approximately 1.8 times that in the Chinese population), the LAR ratios (($\mathrm{LA}R_{\mathrm{IMPT}/\mathrm{PAT}}$) remained highly consistent across the four countries: the mean LAR ratios for the ipsilateral lung and whole lungs ranged from 1.07 to 1.09, and for the esophagus from 1.04 to 1.13 (Figure 4). Paired tests showed that under the linear model, PAT markedly reduced absolute LAR values for the ipsilateral lung and whole lungs in all four countries (*p* < 0.001). Under the mechanistic model, reductions were observed for the ipsilateral lung in France (*p* = 0.001), Sweden (*p* < 0.001), and Germany (*p* = 0.003), and for the whole lungs in France (*p* = 0.046) and Sweden (*p* = 0.014), but not in China (*p* = 0.083 for ipsilateral lung; *p* = 1.000 for whole lungs) or Germany (*p* = 0.317 for whole lungs). For the esophagus, no marked differences were found under either model (all *p* > 0.05).

These results are consistent with the OED findings, suggesting that the LAR ratios (IMPT/PAT) were similar across the four populations. However, this similarity primarily reflects that the ratios are derived from the same OED distributions and should not be overinterpreted as formal population independence. Furthermore, at the cohort level, no signal of increased modeled secondary cancer risk with PAT was observed for the ipsilateral lung or the whole lungs in any of the analyzed scenarios. However, for the esophagus, patient‐level variability (LAR ratios 0.2–1.8) indicates that a uniform benefit cannot be assumed.

## 4 DISCUSSION

Lung cancer is one of the malignancies with the highest incidence and mortality worldwide, and radiotherapy plays a crucial role in its comprehensive treatment. With advances in early screening and multimodality therapy, the long‐term survival of lung cancer patients has continuously improved, making the management of treatment‐related late toxicities increasingly prominent. Once secondary cancer occurs, it is often difficult to treat, with a poor prognosis, severely affecting patients’ quality of life.^23^ Therefore, maximizing the reduction of secondary cancer risk while ensuring primary tumor control is of marked clinical importance. This study found that PAT yielded a modeled secondary cancer risk that was not higher, and even slightly lower, than IMPT in key organs at risk such as the ipsilateral lung, whole lungs, and esophagus. Although the magnitude of relative risk reduction was modest (approximately 7%–8%), considering the enormous population of lung cancer patients, this small relative risk reduction could translate into a considerable absolute benefit—namely, a decrease in the actual number of patients who develop secondary cancers in the future. Furthermore, PAT showed superior dosimetric conformity and lower intermediate‐ to high‐dose volumes, consistent with its risk reduction trend. This study applied the OED–LAR model to lung cancer patients and quantitatively compared the secondary cancer risk between PAT and IMPT. The core finding indicates that PAT did not increase the modeled secondary cancer risk at the cohort level for the lung organs. For the esophagus, the wide range of individual LAR ratios (0.2–1.8) indicates that a uniform benefit across all patients cannot be claimed; rather, it demonstrated a mild and consistent risk reduction trend. These results provide a long‐term safety reference for the clinical application of PAT in lung cancer treatment.

Secondary cancer is one of the key complications affecting the long‐term quality of life of patients after radiotherapy. Previous studies have evaluated the secondary cancer risk of different radiotherapy techniques from multiple perspectives. Fontenot et al.^24^ demonstrated through uncertainty analysis that, despite differences in model assumptions, the conclusion that proton therapy reduces secondary cancer risk compared with photon therapy is robust. Xiang et al.^6^ based on large‐scale population data, further confirmed that the incidence of secondary cancer among patients treated with proton therapy was markedly lower than among those treated with intensity‐modulated photon radiotherapy. The long‐term follow‐up study by Chung et al.^25^ also observed a lower incidence of second malignancies in the proton therapy group compared with the photon group. These studies collectively establish the advantage of proton therapy in reducing long‐term risk. As proton therapy technology continues to evolve, researchers have begun to focus on risk differences among different proton techniques. Paganetti et al.^20^ compared passive scattering and spot‐scanning proton therapy and found that the impact of technical differences on secondary cancer risk exhibited a complex trend, suggesting that technical optimization may further reduce risk. However, the majority of the aforementioned studies have focused on cross‐modality comparisons between protons and photons, or have been limited to a few anatomical sites; systematic data on the direct comparison between the emerging PAT and IMPT in lung cancer, a high‐incidence cancer, are still lacking.

The findings of this study are consistent with recent multi‐site investigations. Johnson et al.^3^ in a study covering six anatomical sites, including head and neck, breast, and mediastinum, similarly observed that the secondary cancer risk ratio ($\mathrm{OE}D_{\mathrm{IMPT}/\mathrm{PAT}}$) was clustered around 1.1, suggesting that the effect size of the risk difference between the two techniques is relatively stable across different anatomical sites. That study also systematically analyzed the influence of model parameters, particularly the repopulation factor R, on risk response: for organs with a high *R* value (> 0.8), such as the lung and esophagus, OED is strongly correlated with mean dose, and changes in dose distribution shape have limited impact on overall risk.^26^ The clustered risk ratios observed in this study for the lung (*R* = 0.83) and esophagus (*R* = 0.81) are highly consistent with the above theoretical expectations, further supporting the biological plausibility of the results.

This study further compared the lifetime attributable risk between PAT and IMPT in the context of four population backgrounds: China, France, Sweden, and Germany. The results showed that, despite differences in baseline cancer risk and life expectancy among countries, the LAR ratios (IMPT/PAT) remained highly consistent across all organs (approximately 1.07–1.09 for ipsilateral lung and whole lungs, and 1.04–1.13 for esophagus), and the LAR ratios (IMPT/PAT) did not show statistically markedly deviation from 1 (*p* > 0.05, data not shown). This finding indicates that the LAR ratios (IMPT/PAT) were similar across the four countries. However, this similarity primarily reflects that the ratios are derived from the same OED distributions; confirming population‐independent risk reduction would require prospective studies with patient cohorts from each country. From a clinical translation perspective, this implies that the potential benefit of PAT in reducing secondary cancer risk can be generalized to lung cancer patient populations in different countries and regions.

This study also highlights the practical value of the OED model in capturing dose distribution differences and converting them into risk comparisons. The repopulation factor (*R*) incorporated in the model is crucial for evaluating the impact of volumetric changes in high‐dose regions. Although PAT may slightly increase the very‐low‐dose irradiated volume, it substantially reduces the intermediate‐ to high‐dose volumes—where the risk contribution per unit dose is higher—so the positive effect dominates. This is likely the primary reason that the risk ratios consistently remain above unity. This mechanistic interpretation also provides a theoretical basis for understanding the impact of different proton techniques on secondary cancer.

Nevertheless, the results of this study must be interpreted with caution. First, it is important to emphasize that the present analysis did not account for secondary neutron dose, which may contribute to secondary malignancy risk and may differ between PAT and IMPT due to distinct delivery characteristics. Therefore, our findings reflect only the in‐field risk attributable to the prescribed therapeutic proton dose and should not be interpreted as representing the total secondary cancer risk. Future studies integrating neutron dose estimates are warranted. Second, the magnitude of risk reduction was small and did not reach statistical significance, suggesting that confirmation through larger sample‐size studies is needed. Third, although several comparisons reached statistical significance, the absolute differences in OED and LAR were modest. For instance, the OED reduction for the ipsilateral lung was approximately 5.9%–6.7%, and the absolute LAR reductions were generally below 0.5 percentage points. These small effect sizes suggest that the observed advantages are of limited clinical significance at the individual patient level. Therefore, our results should be interpreted primarily as evidence that PAT does not confer a higher modeled secondary cancer risk compared with IMPT, rather than as a demonstration of a clinically meaningful superiority. Notably, the mean OED differences between PAT and IMPT were substantially smaller than the standard deviations within each treatment group (e.g., ipsilateral lung OED: 7.94 ± 2.97 vs. 8.44 ± 2.95 cGy). This overlap indicates that the observed differences, even when statistically markedly, are unlikely to be clinically discernible at the individual patient level. More importantly, all model predictions carry inherent uncertainties. As noted by Pfaffenberger et al.^27^ different assumptions about the shape of the dose–response curve can lead to orders‐of‐magnitude differences in absolute risk predictions. Although the model parameters adopted in this study are based on population data fitting, inter‐individual radiobiological heterogeneity was not incorporated. Additionally, the model assumes a constant relative biological effectiveness (RBE = 1.1). Recent simulation studies (e.g., Dasukevich et al.^28^) indicate that incorporating variable RBE models affects the absolute risk estimates, although the direction of the advantage of protons over photons may remain unchanged. For PAT, its ability to optimize LET distributions (Li et al.^29^) may introduce more complex biological effect variables, which represent a frontier direction to be integrated into future risk models.

It is worth emphasizing that, despite the aforementioned uncertainties, the convergence of results from this study with those from previous multi‐center studies provides mutually corroborating evidence for the safety of PAT concerning secondary cancer risk. Similar trends observed across different studies, anatomical sites, and sample sizes indicate that the conclusion that PAT is “non‐inferior or even slightly superior” to IMPT has a certain degree of robustness. However, this robustness may partly stem from the limited differences in dose distribution between the two techniques themselves, as pointed out by Johnson et al.^4^; the OED ratios between IMPT and PAT cluster around 1.1 for most organs, far smaller than the risk difference between protons and photons (which can be up to 10‐fold).

This study has several limitations, including a single‐center retrospective design, limited sample size, and risk prediction based entirely on models rather than long‐term clinical endpoint events. Our cohort had a predominance of male patients (22 males and 3 females), which may limit the generalizability of our findings to female lung cancer patients. Although the risk models incorporate sex‐specific adjustment factors, the small female subsample precludes a robust sex‐stratified analysis. Another important limitation concerns the interpretation of LAR in this older population (median age 65 years). The LAR calculations rely on general population life tables, which may not adequately account for the competing mortality risks associated with primary lung cancer. Patients with lung cancer have a shorter life expectancy than the average population of the same age, and because radiation‐induced secondary cancers typically have a latency of 10–20 years, the LAR estimates may be overestimated. Therefore, our LAR results should be interpreted as modeled projections rather than realized absolute clinical risks. Furthermore, the model relies on a constant RBE value and does not account for its potential spatial variation in vivo, nor does it incorporate possible synergistic effects of concurrent chemotherapy or individual patient genetic susceptibility factors. Future research could proceed in the following directions: first, conducting larger‐scale prospective clinical studies linking precise three‐dimensional dose distributions with long‐term follow‐up outcomes; second, exploring the integration of more complex biophysical variables, such as LET distributions, into the risk model^28^, ^29^; third, advancing the establishment of a multi‐dimensional risk prediction system that integrates dosimetry, biological markers, and clinical endpoints.^30^ Additionally, the risk parameters for the European Union countries used in this study represent population‐averaged estimates for Europe and did not further stratify by sex or age subgroups within each country, which may introduce some uncertainty.

## 5 CONCLUSION

In summary, this study confirms that in lung cancer patients, PAT does not increase the modeled in‐field secondary cancer risk compared with IMPT and suggests a modest, non‐ marked trend toward risk reduction that warrants further investigation. Given the high incidence of lung cancer and the widespread use of radiotherapy, these findings provide long‑term safety evidence supporting the clinical application of PAT as an emerging advanced proton therapy technique that achieves more precise treatment without increasing the patient's secondary cancer risk.

## AUTHOR CONTRIBUTIONS

Study concept and design: Xianliang Wang and Yuankang Xu. Acquisition of data and images, dataset processing: Yuankang Xu, Wenguang Chen, Xianliang Wang, Junxiang Wu, Hao Guo, Feng Yang, Tianyu Zhang, Da Zhang and Xinhui Huang. Analysis and interpretation of data: Yuankang Xu and Wenguang Chen. Manuscript preparation: Yuankang Xu and Xianliang Wang. Writing: Yuankang Xu and Wenguang Chen. Critical revision: Yuankang Xu, Xianliang Wang, Junxiang Wu and Hao Guo. All authors read and approved the final manuscript.

## CONFLICT OF INTEREST STATEMENT

The authors declare that they have no competing interests (financial and non‐financial) in the manuscript.

## ETHICS STATEMENT

This retrospective study was approved by the Ethics Committee for Medical Research and New Medical Technology of Sichuan Cancer Hospital (approval No. KY‐2026‐085‐02, date of approval: June 4, 2026). The requirement for informed consent was waived due to the retrospective nature of the study.

## Data Availability

The datasets generated and/or analyzed during the current study are not publicly available due to patient privacy and confidentiality considerations. De‐identified data may be available from the corresponding author upon reasonable request, subject to institutional data protection policies.
